# Effect of Serum IL-6 Levels on the Progression of Non-Target Lesions in Patients after Coronary Stenting

**DOI:** 10.31083/j.rcm2507234

**Published:** 2024-06-27

**Authors:** Xiang Sha, Wei Wang, Jie Qiu, Ruzhu Wang

**Affiliations:** ^1^Department of Cardiology, The Affiliated Taizhou People’s Hospital of Nanjing Medical University, Taizhou School of Clinical Medicine, Nanjing Medical University, 225300 Taizhou, Jiangsu, China

**Keywords:** interleukin-6, atherosclerotic cardiovascular disease, coronary angiography, coronary stent implantation, non-target lesion progression

## Abstract

**Background:**

percutaneous coronary intervention (PCI) has become the mainstay of treatment for atherosclerotic cardiovascular disease (ASCVD). Inflammatory factors have been shown to be involved in the initiation and progression of ASCVD. After PCI, the persistence of inflammation, especially the inflammation released at the target lesion, may affect the stability of non-target lesion plaques. Interleukin-6 (IL-6) is one of the most common inflammatory factors, however studies about the influence of IL-6 on the progression of non-target lesions (NTLs) of coronary artery are limited. This study investigated whether serum IL-6 levels can affect the progression of NTLs after coronary stent implantation.

**Methods:**

We performed a retrospective cohort study including 441 patients undergoing coronary angiography (CAG) and stent implantation, who had at least one NTL, between January 2019 and December 2021. They underwent followup CAG 9 to 12 months after PCI. Quartile grouping was based on serum IL-6 levels following readmission. The relationship between serum IL-6 levels and the progression of NTLs after coronary stent implantation was analyzed by using logistic regression analysis and restricted cubic spline regression. Predictive value of IL-6 on NTL progression was evaluated using the receiver operating characteristic (ROC) curve.

**Results:**

When compared to the first quartile (Q1) group, the probability of NTL progression was increased in Q2 (adjusted odds ratio (aOR) 3.06, 95% CI 1.29–7.29), Q3 (aOR 3.55, 95% CI 1.52–8.26), and Q4 group (aOR 7.51, 95% CI 3.30–17.05), with a trend test *p* < 0.001. With the increase of IL-6 levels, the risk of progression of NTLs gradually increased, and there was a non-linear relationship between IL-6 and progression of NTLs (*p* < 0.001). The ROC curve showed that the critical value of the serum IL-6 level was 12.652 pg/mL (area under the curve is 0.673, sensitivity is 54.5%, specificity is 70.9%, *p* < 0.05).

**Conclusions:**

A high serum IL-6 level is an independent risk factor for the progression of NTLs after coronary stent implantation, and has certain predictive value for the progression of NTLs.

## 1. Introduction

atherosclerotic cardiovascular disease (ASCVD) has become the leading cause of death and disease burden in China and worldwide. Coronary artery disease can be treated with percutaneous coronary intervention (PCI). The progression of native coronary atherosclerotic lesions continues to pose a significant clinical challenge following PCI [1]. Despite control of ASCVD risk factors, nearly 60% of patients still experience progression of coronary artery plaques, accompanied by a significant increase in the risk of cardiovascular events [2].

Epidemiological and biological studies show that inflammation is involved in the initiation and progression of coronary atherosclerosis. It has recently been demonstrated that nearly all recognized mechanisms related to the formation, advancement, susceptibility, and rupture of atherosclerotic plaques are linked to the activation and persistence of inflammatory processes [3]. Circulating inflammatory biomarkers, including hypersensitive C-reactive protein (hsCRP) and interleukin-6 (IL-6), are independently associated with cardiovascular events [4]. Studies have showed that innate immune pathways targeting IL-1β to IL-6 further reduce cardiovascular events in patients treated with statins [5]. Therefore, the assessment of “inflammatory risk” has been proposed as a reasonable strategy to strengthen risk prediction and guide the deployment of cost-effective and precise treatment for cardiovascular disease prevention.

One study found that after placement of a stent in target lesions, other coronary artery vessels (non-target lesions) showed progression of lesions [1]. Similarly, animal studies showed that after coronary stent implantation, other vascular plaque load significantly increased, and serum levels of acute-phase inflammatory proteins significantly increased [6]. Currently, there is little research on the relationship between serum IL-6 levels and the progression of non-target lesions (NTLs) after coronary stent implantation. Therefore, in the present study, a patient cohort who underwent PCI with stent implantation and subsequent follow-up coronary angiography (CAG) was assessed to explore the potential impact of serum IL-6 levels on the risk of coronary artery plaque progression. This information will contribute to a better understanding of the impact of inflammation on the progression of non-target lesions in patients after coronary stenting.

## 2. Materials and Methods

### 2.1 Study Design and Patients

A retrospective study was conducted at the Taizhou People’s Hospital affiliated with Nanjing Medical University, China. This retrospective analysis included all patients undergoing CAG and stent implantation on initial admission, who had at least one NTL, between January 2019 and December 2021. Followup CAG was performed 9 to 12 months after stent placement. Quartile grouping was based on serum IL-6 levels at the time of readmission.

The exclusion criteria were: (1) Patients with incomplete clinical data or imaging data; (2) Fractionated elective coronary intervention of multiple coronary artery lesions; (3) Patients readmitted for diagnosis of acute coronary syndrome; (4) Previous cardiac surgery such as coronary artery bypass grafting and other PCI; (5) Patients with immune system diseases; (6) Patients with active infections; (7) Patients with malignancies; (8) Patients administered anti-inflammatory or immuno-suppressive drugs, with the exception of aspirin.

The electronic medical record system of the Taizhou People’s Hospital affiliated with Nanjing Medical University was reviewed to collect clinical data at readmission, including age, gender, body mass index (BMI); risk factors for coronary heart disease, including hypertension, diabetes, smoking history and postoperative smoking; Laboratory test results, including routine blood tests, renal function, blood lipid levels, glycosylated hemoglobin (HbA1c), IL-6, neutrophil gelatinase associated lipid transport protein (NGAL), lipoprotein-associated phospholipase A2 (LP-PLA2); Echocardiography results, including left ventricular end diastolic (LVED) volume and left ventricular ejection fraction (LVEF); and the imaging results of the two followup CAGs.

Blood samples were obtained upon readmission in order to examine the prognostic significance of IL-6. Venous blood (5 mL) was collected in the morning after overnight fasting, and the serum fraction was separated by centrifugation. The serum IL-6 were detected by chemiluminescence immunoassay using Quantitative Assay Kits (mindray, Shenzhen, China). The results were expressed in pg/mL.

All participants received standard medication therapy including statins, aspirin, β-blockers, angiotensin converting enzyme inhibitors/angiotensin receptor blockers, calcium channel blockers, and oral nitrates, both after stent implantation and throughout the duration of the study. None of the patients were administered anti-inflammatory or immuno-suppressive drugs, with the exception of aspirin.

This study was approved by the Ethics Committee of the Taizhou People’s Hospital affiliated with Nanjing Medical University, with ethics approval number KY2022-198-01.

### 2.2 Definitions

The target vessel was defined as the entire major coronary vessel proximal and distal to the target lesion including proximal and distal branches and the target lesion itself. The NTL refers to a stenotic lesion found in a non-target coronary vessel, unrelated to ischemic symptoms or positive functional ischemic test results [7]. Lesion progression was determined by the increase in percent diameter stenosis, which was calculated using percent diameter stenosis at the second CAG minus that at the first CAG. The progression of NTL was defined based on the presence of any of the following criteria: (1) an increase of more than 10% diameter reduction of a pre-existing diameter stenosis that was ≥50%; (2) an increase of more than 30% diameter reduction of a pre-existing stenosis that was <50%; (3) the development of a new stenosis with a ≥30% reduction in the diameter of a segment that was normal on the first angiogram, or the progression of any lesion to total occlusion on follow-up CAG. When at least one lesion showed progression, the patient was considered to be a progressor. However, in this study, we only included the patients who met one of the first two criteria.

### 2.3 The Process of CAG and Coronary Stenting and Angiographic Follow-up

Coronary angiography, interventional therapy, and postoperative reports were performed by two experienced cardiologists. The target lesion (TL) was treated using standard interventional techniques with the implantation of drug-eluting stents. In our study, we utilized two types of drug-eluting stents, the sirolimus-eluting stent and the paclitaxel-eluting stent. To maintain an activated clotting time of 250 seconds, pre-procedural intravenous heparin was administered. Additionally, all patients were prescribed a daily dose of at least 100 mg of aspirin for a minimum of one year, as well as a loading dose of 300 mg of clopidogrel followed by a daily dose of 75 mg or a loading dose of 180 mg of Ticagrelor followed by 90 mg bid until the angiographic follow-up. A repeat follow-up coronary angiography was scheduled 9–12 months after coronary stenting.

### 2.4 Quantitative Coronary Arteriography Analysis

The assessment of the progression of NTLs was conducted using a study protocol consisting of two consecutive quantitative CAG. The first CAG was performed upon initial admission, while the second CAG was conducted during the follow-up period. Two experienced cardiologists performed coronary angiography by using the Judkins method to perform left and right coronary angiography and multi-axial imaging. Two end-diastolic angiographic projection angles were captured, in cases of no foreshortening or overlap between segments. For CAG, 5F Tiger catheters were the first choice, and each patient was analyzed using Philips Xcelera software (Philips, Amsterdam, The Netherlands) to determine the quality of the quantitative coronary angiography (QCA). Among the measurements were the diameter of the reference vessel, the minimum diameter of the lumen, the length of the lesion, and the diameter stenosis. It was decided to average values obtained from two different angles in the end diastolic phase in order to reduce angiography errors. The first QCA result and the repeat follow-up coronary angiography were compared to evaluate the progression of NTLs.

### 2.5 Main Outcome Measures

The primary outcome measured in the present study was the progression of non-target lesions in patients after coronary stenting.

### 2.6 Statistics Analysis

All the data was stored and processed in R software (R version 4.1.0, R Development Core Team, R Foundation for Statistical Computing, Wien, Austria). The continuous variables were described as mean and standard deviation (normal distribution) or as median and quartiles (skewed distribution). The comparisons between groups were tested by analysis of variance or Kruskal-Wallis test. The IL-6 level was divided into 4 groups based on quartiles. The Logistic regression models were used to estimate the odds ratios (ORs) and their 95% confidence intervals (CIs) between IL-6 and NTL progression. Variables which were statistically associated with IL-6 in univariate analyses were treated as potential confounders and adjusted in the multivariable Logistic models. Restricted cubic spline regression was used to explore the nonlinear correlation between IL-6 and the risk of NTL progression. The receiver operating characteristic (ROC) curve was drawn and area under the curve (AUC) was estimated for evaluating the accuracy of IL-6 levels in predicting NTL progression. The optimal threshold was estimated based on the maximum Youden index, then the sensitivity and specificity under this threshold were calculated. *p* values less than 0.05 was considered statistically significant.

## 3. Results

Between January 2019 and December 2021, a total of 441 patients were included in this study. They underwent a follow-up angiogram after a mean period of 10.5 months. The median IL-6 level at readmission was 8.78 pg/mL (interquartile range (IQR) 4.6, 15.33). Patients were categorized into 4 groups according to quartiles of IL-6 levels: the first quartile (Q1) group (≤4.6 pg/mL), second quartile (Q2) group (4.6–8.78 pg/mL), third quartile (Q3) group (8.78–15.33 pg/mL) and fourth quartile (Q4) group (≥15.33 pg/mL). The number of cases of progression of NTLs in each group was 9 (8.11%), 24 (21.82%), 26 (23.64%), and 42 (38.18%), respectively.

The demographic and clinical data of patients were presented in Tables 1,2. The incidence of diabetes (40.9%) was higher in the Q4 group than in patients in the other groups (*p* = 0.018). There were no statistically significant differences in age, gender, BMI, LVED, LVEF, arterial pressure and resting heart rate, hypertension, previous smoking history, smoking after PCI, previous myocardial infarction, three vessel lesions, and medication intake among the different groups (*p* > 0.05). Similarly, hsCRP was not significantly different among the four groups (*p* > 0.05). The apolipoprotein A/B (APOA/B) of the Q1 group was significantly higher than that of the other groups (*p* = 0.032), while the HbA1c of Q4 group was significantly higher than that of the other groups (*p* = 0.008). No significant differences in red blood cell count, hemoglobin, platelet count, ratio of neutrophils to lymphocytes, total cholesterol, triglycerides, high-density lipoprotein cholesterol, low-density lipoprotein cholesterol (LDL-C), lipoprotein A, blood homocysteine, fructosamine, urea nitrogen, creatinine, uric acid, NGAL, and LP-PLA2 were identified among the different groups (*p* > 0.05).

**Table 1.** **Baseline characteristics**.

		Q1	Q2	Q3	Q4	*p*
n		111	110	110	110	
Age, years		63.01 (11.54)	63.19 (10.69)	63.21 (11.08)	63.51 (10.57)	0.99
Sex, n (%)						
	Female	25 (22.5)	23 (20.9)	14 (12.7)	29 (26.4)	0.084
	Male	86 (77.5)	87 (79.1)	96 (87.3)	81 (73.6)	
BMI, ${\mathrm{kg/m}}^{2}$		26.94 (15.54)	24.79 (2.88)	27.32 (24.19)	25.03 (3.45)	0.455
Hypertension, n (%)		78 (70.3)	67 (61.5)	70 (63.6)	73 (66.4)	0.551
Diabetes, n (%)		29 (26.1)	27 (24.8)	27 (24.5)	45 (40.9)	0.018
Smoking history, n (%)		34 (30.6)	41 (37.3)	48 (43.6)	46 (41.8)	0.194
Postoperative smoking, n (%)		12 (10.8)	18 (16.4)	20 (18.2)	20 (18.2)	0.387
Previous history of myocardial infarction, n (%)		32 (28.8)	42 (38.2)	31 (28.2)	43 (39.1)	0.242
Postoperative medication for coronary stent implantation						
	Aspirin, n (%)	107 (98.2)	106 (96.4)	109 (99.1)	109 (99.1)	0.384
	Anticoagulants, n (%)	0 (0.0)	5 (4.6)	1 (0.9)	1 (0.9)	0.662*
	Clopidogrel, n (%)	68 (63.0)	79 (72.5)	80 (72.7)	64 (58.2)	0.053
	Ticagrelor, n (%)	43 (39.8)	29 (26.6)	30 (27.5)	45 (40.9)	0.034
	Statin therapy, n (%)	107 (99.1)	107 (98.2)	110 (100.0)	109 (99.1)	0.566
	β-blocker, n (%)	79 (73.1)	83 (76.1)	71 (64.5)	78 (71.6)	0.274
	ACEI/ARBs/ARNI, n (%)	79 (74.5)	79 (73.8)	83 (75.5)	80 (73.4)	0.987
SBP, mmHg		129.86 (23.59)	132.85 (20.57)	131.66 (14.62)	132.90 (18.79)	0.628
DBP, mmHg		83.58 (20.01)	83.26 (12.73)	79.73 (10.31)	81.81 (13.30)	0.187
Resting heart rate, bpm		73.55 (23.79)	73.82 (17.60)	73.62 (12.57)	72.10 (16.37)	0.89

*Fisher’s exact test. BMI, body mass index; ACEI, angiotensin-converting enzyme inhibitor; ARBs, angiotensin receptor blockers; ARNI, angiotensin receptor-enkephalin inhibitor; SBP, systolic blood pressure; DBP, diastolic blood pressure.

**Table 2.** **The result of the clinical examination and the blood tests**.

	Q1	Q2	Q3	Q4	*p*
n	111	110	110	110	
Red blood cell count (× ${\mathrm{10}}^{9}$/L)	4.52 [4.04, 4.82]	4.42 [4.07, 4.84]	4.46 [4.14, 4.81]	4.50 [4.12, 4.85]	0.951
Hemoglobin (g/L)	135.00 [120.00, 145.50]	136.00 [123.25, 147.00]	136.50 [124.25, 147.00]	137.00 [123.50, 146.00]	0.714
White blood cell count (× ${\mathrm{10}}^{9}$/L)	5.74 [4.95, 7.03]	5.57 [4.93, 6.90]	5.77 [4.79, 6.78]	5.80 [4.96, 6.72]	0.89
Platelet count (× ${\mathrm{10}}^{9}$/L)	170.00 [119.50, 212.00]	172.00 [135.25, 212.00]	172.50 [141.50, 207.00]	175.50 [127.00, 203.25]	0.96
NLR	2.26 [1.73, 3.09]	2.33 [1.81, 3.06]	2.58 [2.01, 3.30]	2.57 [1.86, 3.54]	0.112
Total cholesterol (mmol/L)	3.14 [2.60, 3.58]	3.12 [2.76, 3.65]	3.26 [2.61, 3.75]	3.18 [2.80, 3.87]	0.663
Triglyceride (mmol/L)	1.17 [0.98, 1.90]	1.38 [0.96, 1.85]	1.13 [0.83, 1.71]	1.30 [1.02, 1.85]	0.093
HDL-C (mmol/L)	1.02 [0.85, 1.27]	0.99 [0.87, 1.18]	1.00 [0.87, 1.15]	1.03 [0.84, 1.19]	0.652
LDL-C (mmol/L)	1.82 [1.46, 2.14]	1.83 [1.62, 2.36]	1.88 [1.44, 2.26]	1.84 [1.53, 2.26]	0.453
APOA/B	2.00 [1.65, 2.50]	1.87 [1.47, 2.24]	1.89 [1.48, 2.27]	1.88 [1.49, 2.37]	0.032
Lipoprotein (a) (mmol/L)	174.00 [67.00, 381.00]	218.50 [79.25, 473.00]	231.00 [102.00, 382.10]	220.50 [86.00, 428.85]	0.415
HCY (µmol/L)	11.90 [9.30, 14.84]	11.10 [8.90, 13.82]	12.35 [10.35, 15.43]	11.42 [9.03, 14.55]	0.121
HbA1c (%)	6.40 [5.90, 7.00]	6.20 [5.80, 6.80]	6.25 [5.80, 6.77]	6.65 [6.00, 7.90]	0.008
Fructosamine (mmol/L)	204.50 [183.25, 243.50]	207.00 [188.00, 233.75]	206.50 [183.00, 230.00]	213.50 [188.00, 242.50]	0.548
Creatinine (µmol/L)	69.40 [56.20, 79.30]	65.30 [53.52, 76.22]	69.40 [55.52, 78.18]	67.80 [55.50, 81.00]	0.444
Oleuropein (mmol/L)	5.67 [4.69, 7.11]	5.43 [4.87, 6.69]	5.86 [4.81, 7.29]	5.38 [4.61, 6.70]	0.347
Ursolic acid (µmol/L)	322.00 [275.00, 401.50]	344.50 [281.25, 394.75]	326.00 [262.00, 384.00]	335.00 [287.00, 400.00]	0.643
NGAL (ng/mL)	137.75 [73.16, 214.38]	126.31 [74.90, 178.30]	125.63 [69.76, 219.53]	124.28 [71.33, 193.87]	0.606
LP-PLA2 (ng/mL)	150.49 [113.12, 193.44]	154.72 [112.11, 197.96]	155.04 [114.25, 204.96]	137.24 [107.91, 179.38]	0.285
hsCRP (mg/L)	0.53 (0.31)	0.58 (0.29)	0.58 (0.47)	0.62 (0.37)	0.658
Triple vessel lesion, n (%)	34 (31.8)	35 (33.7)	30 (28.6)	42 (40.8)	0.293
LVED (mm)	49.54 (4.20)	49.03 (5.13)	49.88 (4.68)	49.90 (5.00)	0.559
LVEF (%)	66.02 (8.44)	64.53 (8.68)	65.46 (8.34)	64.90 (9.48)	0.633

NLR, neutrophil to lymphocyte ratio; HDL-C, high-density lipoprotein cholesterol; LDL-C, low-density lipoprotein cholesterol; APOA/B, apolipoprotein A/apolipoprotein B; HCY, homocysteine; NGAL, neutrophil gelatinase associated lipid transport protein; LP-PLA2, lipoprotein-associated phospholipase A2; LVED, left ventricular end diastolic; LVEF, left ventricular ejection fraction; HbA1c, glycosylated hemoglobin; hsCRP, hypersensitive C-reactive protein.

Multivariable logistic regression included variables with a *p* value of <0.10 in the univariate analysis. After adjusting for gender, diabetes, clopidogrel, ticagrelor, triglyceride, APOA/B, HbA1, compared to the reference level (Q1), the probability of NTL progression was increased in Q2 (adjusted odds ratio (aOR) 3.06, 95% CI 1.29–7.29), Q3 (aOR 3.55, 95% CI 1.52–8.26), and the Q4 group (aOR 7.51, 95% CI 3.30–17.05) (Table 3). With the increase of IL-6 levels, the risk of NTL progression showed an increasing trend, *p* < 0.001.

**Table 3.** **The odds ratios between IL-6 level and the progression of NTLs**.

	Crude model	Adjusted model
IL6	OR (95% CI)	OR (95% CI)
Continuous	1.04 (1.02–1.07)	1.04 (1.02–1.07)
Q1	Ref	Ref
Q2	3.16 (1.40–7.17)	3.06 (1.29–7.29)
Q3	3.51 (1.56–7.89)	3.55 (1.52–8.26)
Q4	7.00 (3.20–15.31)	7.51 (3.30–17.05)
*p* for trend	<0.001	<0.001

Adjusted model, adjusting for gender, diabetes, clopidogrel, ticagrelor, triglyceride, APOA/B, HbA1 (Only variables with *p* < 0.10 on univariate analysis were displayed). IL-6, interleukin-6; NTLs, non-target lesions; OR, odds ratio; HbA1, glycosylated hemoglobin; APOA/B, apolipoprotein A/apolipoprotein B.

**Fig. 1.**
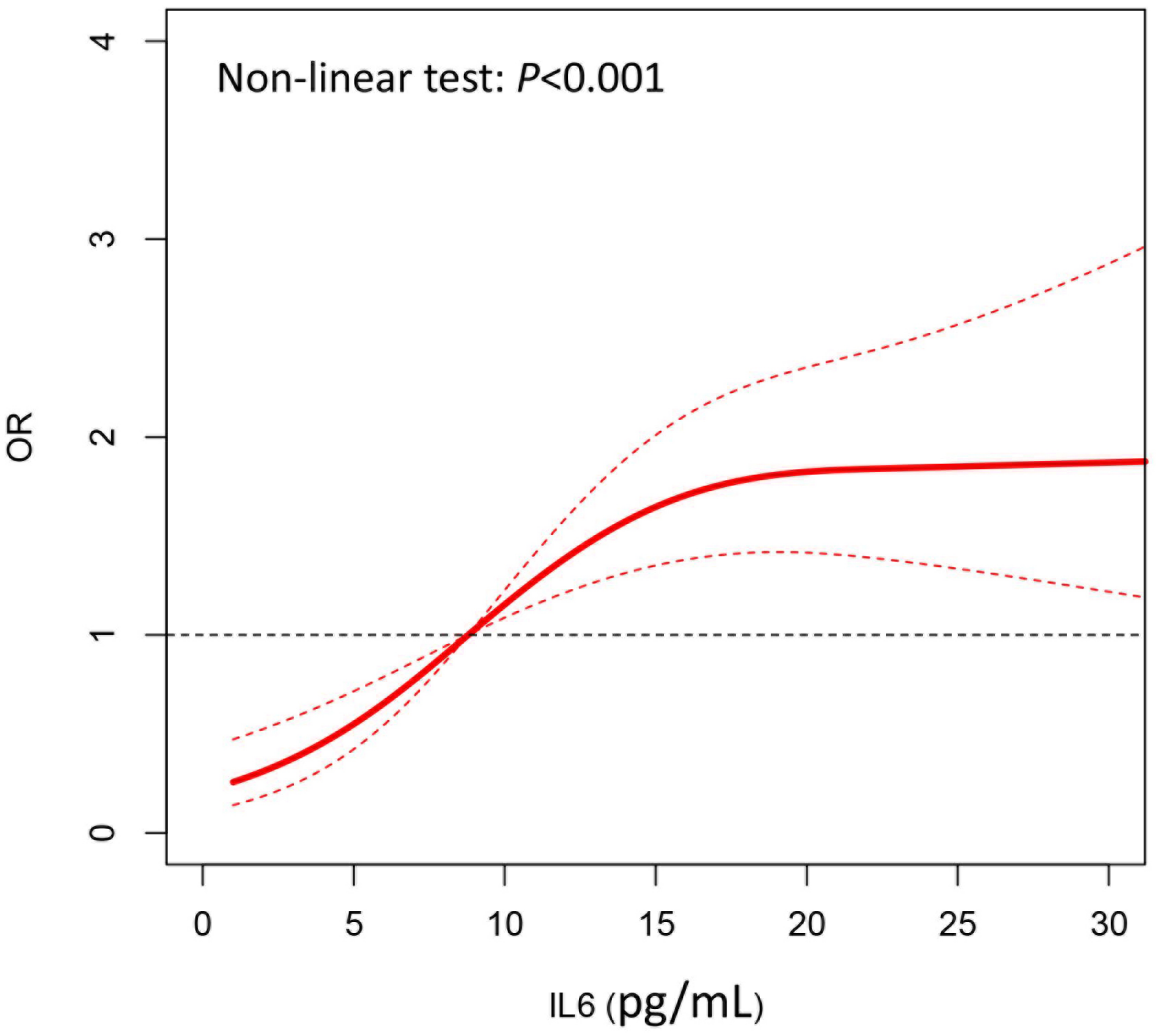
**Cubic spline for the association between IL-6 and odds of NTL progression.** IL-6, interleukin-6; NTL, non-target lesion; OR, odds ratio.

The restricted cubic spline curve showed that after adjustment for gender, diabetes, clopidogrel, ticagrelor, triglyceride, APOA/B, and HbA1, the risk of progression of NTL of coronary artery gradually increased with the increase of IL-6 level, and there exited a non-linear relation between IL-6 and progression of NTLs (*p* < 0.001) (Fig. 1). After the level of IL-6 increased to a certain extent (>20 pg/mL), the risk of progression of NTLs no longer increased.

**Fig. 2.**
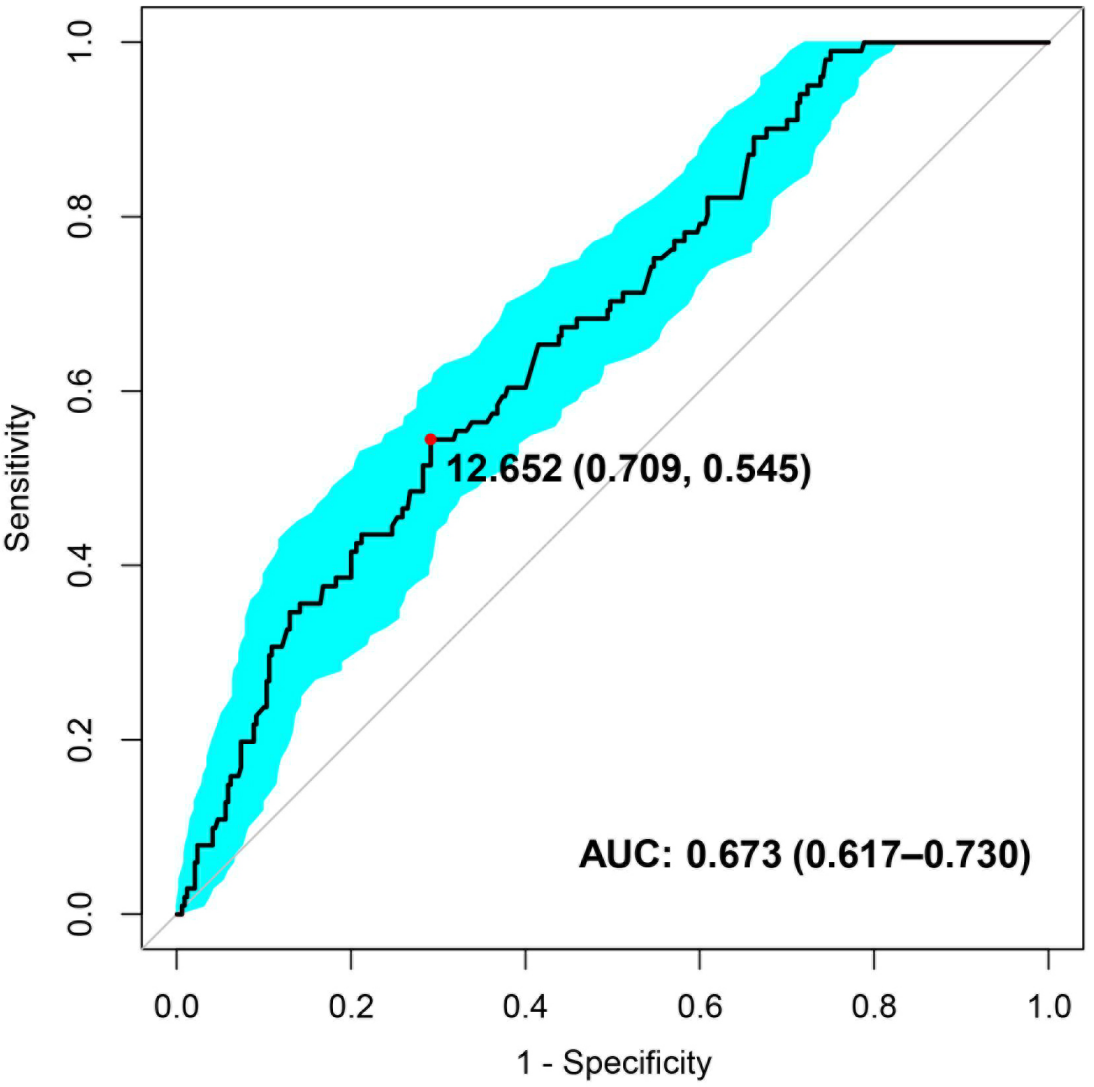
**Receiver operating characteristic (ROC) curves of the predictive ability of IL-6 regarding the progression of NTLs. **IL-6, interleukin-6; NTLs, non-target lesions; AUC, area under the curve.

ROC curve analysis showed that the AUC of serum IL-6 levels predicting the progression of NTLs after coronary stent implantation was 0.673 (*p* < 0.05), with a 95% CI of 0.617–0.730 (Fig. 2). When the cut-off point for IL-6 detection was >12.652 pg/mL as the diagnostic point for NTL progression, the sensitivity and specificity of IL-6 in predicting NTL progression were 54.5% and 70.9%, respectively.

## 4. Discussion

In this study, we found that a high serum IL-6 level was an independent risk factor for the progression of non-target lesions in patients after stent implantation, and might be an independent predictor in evaluating the progression of NTLs.

Low-grade chronic inflammation is a critical process in the pathogenesis and development of ASCVD [8]. Coronary stent implantation is currently considered a safe and widely accepted strategy to treat severe coronary artery stenosis. However, in the context of optimizing secondary preventive drug treatment and effectively controlling risk factors, a residual risk of cardiovascular adverse events persist [9]. It has been found that in rabbit models of atherosclerosis, stent implantation caused an acute phase response and systemic inflammation. Many inflammatory factors, including IL-6, are significantly elevated up to one month following a PCI. Plaque burden in non-target lesions was significantly increased 12 weeks after stent implantation [6]. IL-6 is regarded as an upstream inflammatory cytokine, which is central to the inflammatory cascade, and is also a critical mediator in the inflammatory process, and is involved in atherogenesis and destabilization of atherosclerotic plaque [10]. IL-6 is involved in the initiation and progression of atherosclerosis, which actives inflammatory reactions and regulates oxidative stress by stimulating the liver to synthesize acute phase reactants, activating endothelial cells, promoting lymphocyte proliferation and differentiation, enhancing blood coagulation, which then accelerates lipid deposition and the formation of foam cells [11]. When compared with the downstream inflammatory factors C-reactive protein (CRP) and fibrinogen, IL-6 is more significantly associated with the incidence of cardiovascular events [12].

Based on previous studies, we further focused on patients who underwent CAG following PCI to clarify the progression of non-interference vascular lesions and to obtain more direct clinical evidence for the correlation between IL-6 and the progression of coronary artery plaques. In order to make the research subjects more homogeneous and comparable, we screened and excluded patients diagnosed with acute coronary syndrome, since the acute coronary syndrome is characterized by systemic inflammation. Follow-up CAG was performed 9 to 12 months after PCI. In order to make the observation time of lesions more consistent, this study excluded patients who were readmitted to the hospital for a followup CAG within 9 months after surgery due to recurrent chest tightness and pain or for other reasons.

The 441 patients in this study who underwent coronary stent implantation had relatively complete secondary prophylactic drug treatment. Although patients had lower HDL-C levels of 1.80 mmol/L (1.50–2.18), and traditional risk factors such as blood pressure, blood sugar, and BMI were also well controlled, 22.9% of patients still experienced progression of NTLs 9 to 12 months after PCI. In the comparison of clinical and examination data between groups, we found statistical differences in diabetes, APOA/B and HbA1c. Similar to our study, Niculet *et al*. [13] confirmed that IL-6 was associated with lipid disorders and abnormal glucose metabolism. Multiple studies have confirmed that APOA/B has a stronger independent correlation with vascular risk and is a better risk predictor than traditional lipid indicators such as LDL-C [14]. Intravascular ultrasound studies in patients after PCI have found that in a subgroup of patients with type 2 diabetes, when HbA1c is >6.5%, the patients’ NTLs progressed more rapidly, and the lipid content within the coronary plaque was also significantly increased [15]. There is also definite evidence that hsCRP could predict the risk of future cardiovascular events, independent of traditional risk factors [16]. However, there was no significant statistical difference in hsCRP between each of the quartile groups in our study. The reported atherogenic effect of hsCRP in previous studies is still controversial [17]. The reason for the lack of difference of hsCRP between groups might be the use of statins in the post-operative period. Several studies have shown that statin therapy lowered CRP levels independent of its lipid-lowering effects [18, 19]. After adjustment for diabetes mellitus, APOA/B, and HbA1, we found that compared with the Q1 group, the risk of NTL progression in the other three groups was increased by 3.06, 3.55 and 7.51, respectively, with statistically significant differences between the groups. Furthermore, we found a non-linear relationship between IL-6 and the progression of NTLs using non-restrictive cubic splines. With the increase of IL-6 level, the risk of NTL progression gradually increased, and clear border effects were present. After the level of IL-6 increased to a certain extent (>20 pg/mL), the risk of NTL progression in coronary arteries no longer increased, which was in line with the rule of thumb.

A correlation has been demonstrated between IL-6 levels and endothelial dysfunction and subclinical atherosclerosis [20]. Subirana *et al*. [21] found that after adjustment for age and gender, the serum IL-6 level was correlated with the incidence of morbidity in coronary heart disease (CHD). In multivariate analysis, higher levels of serum IL-6 was significantly associated with major adverse cardiovascular events after adjusting for other factors [22]. Multiple prospective studies and meta-analyses have confirmed that, there was an association between IL-6 levels and future risks of cardiovascular disease [8]. In conclusion, the level of IL-6 has clinical value in various stages of the development of CHD, and can predict long-term risk. Our study is the first to estimate the effect of IL-6 on the progression of NTLs. Although this was a retrospective study, strict inclusion and exclusion criteria were performed to control for confounding factors. Our study further confirmed that the high level of serum IL-6 was an independent risk factor for the progression of NTLs after coronary stent implantation, had a certain predictive value for the progression of NTLs, and broadened the clinical adaptation of IL-6 in various populations of CHD.

There were several limitations in this study. We were limited by the use of the lectronic medical record system, which creates the possibility of confounding bias because of unmeasured confounding factors. This observational study cannot confirm whether the association between exposure and outcome is causal, and further clinical randomized controlled trials are needed. We only conducted a single measurement for IL-6, if IL-6. Serial measurements of these values will be more valuable to study their clinical effects. Lastly, since intracoronary imaging was only performed in some patients, the nature, location, and classification of plaque lesions were not included in the analysis.

## 5. Conclusions

Increased levels of serum IL-6 are independent risk factors for the progression of NTLs after coronary stent implantation. IL-6 has a certain predictive value for the progression of NTLs and provides guidance for secondary prevention in the management of coronary heart disease. However, further research is needed to better determine the value of IL-6 following PCI.

## Data Availability

The original contributions presented in the study are included in the article. Further inquiries can be directed to the corresponding author.
